# Arene diazonium saccharin intermediates: a greener and cost-effective alternative method for the preparation of aryl iodide

**DOI:** 10.3906/kim-2002-26

**Published:** 2020-04-01

**Authors:** Lia ZAHARANI, Nader GHAFFARI KHALIGH, Zohreh SHAHNAVAZ, Mohd RAFIE JOHAN

**Affiliations:** 1 Nanotechnology & Catalysis Research Centre, Institute of Postgraduate Studies, University of Malaya, Kuala Lumpur Malaysia

**Keywords:** Aromatic iodide, one-pot diazotization-iodination, saccharin, waste prevention

## Abstract

In the current protocol, the arene diazonium saccharin derivatives were initially produced from various substituted aromatic amines; subsequently, these intermediates were treated with a greener organic iodide for the preparation of the aryl iodide. We tried to choose low-cost, commercially available, biodegradable, recoverable, ecofriendly, and safe reagents and solvents. The arene diazonium saccharin intermediates could be stored in the liquid phase into a refrigerator for a long time with no significant loss activity. The outstanding merits of the current protocol (a) included the partial recovering of saccharin and tetraethylammonium salt, (b) reduce the use of solvents and the reaction steps due to eliminating separation and purification of intermediates, (c) good yield of the sterically hindered substrates, and (d) avoid the generation of heavy metal or corrosive waste.

## 1. Introduction

In the course of our research regarding the synthesis of aromatic iodides through the Sandmeyer reaction or its alternatives, we required an efficient, low cost, and sustainable method for accessing relative stable arene diazonium salts. The most arene diazonium salts are often prepared in situ due to their instability and explosive risk [1]. It is clear that the anion nature can influence in situ the generation of the arene diazonium salts [2], and facilitated the preparation of aryl iodides. The preparation of relative stable benzenediazonium tetrafluoroborates was reported in ethanol using HBF4 and isoamyl nitrite [3]. Most of the reported chemicals and reagents are expensive, toxic, nonbiodegradable, unrecoverable, and metal-containing, so we looked for a cheap, nontoxic, recoverable, biodegradable, and biocompatible reagent that show high efficiency under mild conditions.

In continuation of our previous work [4] and application of saccharin [5–7], we encouraged to investigate the potential of saccharin for the synthesis of the aryl iodides through in situ formation the arene diazonium saccharin salts which to the best of our knowledge has not been presented to date. According to the current strategy, aryl iodides are formed from diazotization of in situ generated arene diazonium salts which are in turn formed from aniline derivatives and tert-butyl nitrite (TBN) in the presence of saccharin. The intermediates are not isolated and purified in the current protocol which lead to minimizing the solvent waste, and energy efficiency is enhanced by the reaction performance at room temperature and shorter reaction times.

## 2. Results and discussion

TBN and tetraethylammonium iodide (TEAI) were preferred as a nitrating agent and iodide precursors due to their unique properties like safe handling, metal-free, inexpensive, and commercial availability [8,9].

Based on our previous works [4,5], the glacial acetic acid and TBN together with Sac–H were slowly stirred in ethanol at low temperatures (an ice bath). After 5 min, aniline 1a was slowly added at the same temperature. After completion of the first step (monitored by a colour test of azo coupling with 2-naphthol), the generated t -BuOH and unreacted TBN were removed under vacuum by a rotary evaporator. Then, the TEAI solution was added into the stirring intermediate 2a at one time and final product 3a was purified by the flash chromatography (Scheme 1).

**Sceheme 1:**
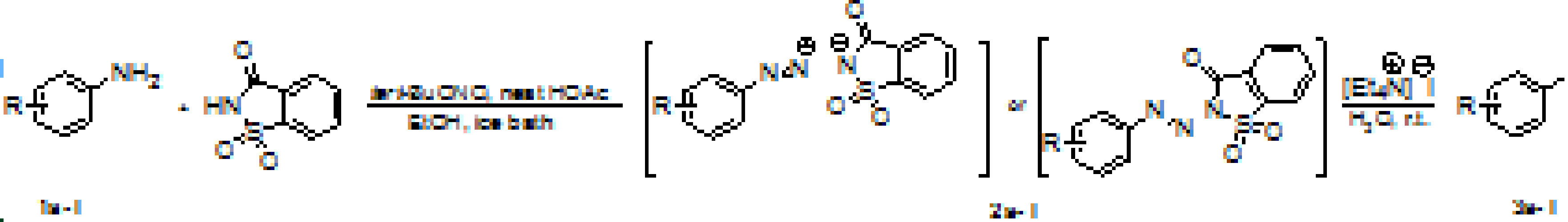
Preparation of aryl iodides using TBN= saccharine, and TEAI.

The scope and generality of current methodology was evaluated through transformation of a variety of aniline derivatives into the corresponding iodides using the current protocol. The aryl iodides bearing electrondonating and electron-withdrawing groups were obtained from the corresponding anilines in good to excellent yields (Table 1). However, the steric hindered anilines viz. 2,6-dimethylaniline, 2,6-diethylaniline, and 2,6-diisopropylaniline 3(d-f) were isolated in 67%, 40%, 42% yields, respectively as expected (Table 1, entry 4–6) [6]. All known products showed an identical melting point and NMR spectra to those in the literature (See Supplementary Material) [4].

**Table 1:** Synthesis of aryl iodides through in situ formation of the arene diazonium saccharin intermediates.^a^

Entry	Ar-NH_2_ 1(a-l)	Product 3(a-l)	Yield (%)b	Melting point (°C)	
				Found	Reported [4]
1	C_6_H_5^-^_	A	80	Oil	Oil
2	4-CH_3_O-C_6_H_4^-^_	B	72	50–51	52–54
3	4-CH_3^-^_C_6_H_4^-^_	C	78	Oil	Oil
4	2,6-(CH_3_)_2_-C_6_H_3^-^_	D	67	Oil	Oil
5	2,6-(CH_3_CH_2_)2-C_6_H_3^-^_	e	40	Oil	Oil
6	2,6-[(CH_3_)2CH]_2_-C_6_H_3^-^_	f	42	Oil	Oil
7	4-Cl-C_6_H_4^-^_	g	79	54–55	55–56
8	2-Cl-C_6_H_4^-^_	h	77	Oil	Oil
9	4-Br-C_6_H_4_	i	80	88–90	87-88
10	4-NO_2_-C_6_H_4^-^_	j	82	168–169	170–172
11	2-NO_2_-C_6_H_4^-^_	k	78	51–52	51–53
12	4-morpholinoaniline	l	70	158–159	159–161

aReaction conditions: TBN (0.30 mL, ~2.3 mmol), aniline derivatives 1(a-l) (2.0 mmol), glacial acetic acid (0.12 mL, 2.1 mmol), Sac-H (0.37 g, 2.0 mmol), TEAI (0.52 g, 2.0 mmol), solvent (H_2_O/EtOH 1:1 v/v, 5 mL); total reaction time (2 h). ^b^Isolated yield.

Complete or partial recovering of the catalyst and reagents are very important in the industrial processes due to minimizing the waste, as well as lower raw material, energy, and waste disposal costs. According to the reported procedure in the literature [6], the saccharin and tetraethylammonium chloride (TEAC) could be recovered in the range of 77%–72% and 68%–64% yield, respectively.

The possible reaction of aniline and saccharin was investigated by a control experiment under optimal conditions. No 3-amino-1, 2-benzisothiazole-1, 1-dioxide were detected by LC-MS analysis.

Another experiment was performed to study the stability of the arene diazonium intermediate. After the first step, the intermediate 2a can be isolated by removing the excess TBN and t -BuOH under vacuum and stored in a refrigerator (4°C). After four days, the stored arene diazonium 2a was treated with a TEAI solution which afforded iodobenzene (3a) in a 74% yield.

A proposed mechanism is illustrated in Scheme 2 [6]. Saccharin and tert-butyl nitrite generate the nitroso (^+^N=O ↔ N≡O^+^) in the first step, which can act as a mild electrophile. The reaction of nitroso and arylamine will form arene diazonium saccharinate [ArN_2_ ]^+^[Sac]^-^ or arenediazosaccharine adduct Ar–N=N–Sac. High stability of intermediate assigned to the inductive and resonance effects of the carbonyl and sulfonyl groups on saccharin moiety. In the second step, [ArN_2_ ]^+^[Sac]^-^ salt or Ar–N=N–Sac adduct will participate in an electrophilic substitution reaction with TEAI, which finally affords the corresponding aryl iodides. Our group is working to investigate the detailed processes of reaction mechanism based on 2 routes A and B.

**Sceheme 2:**
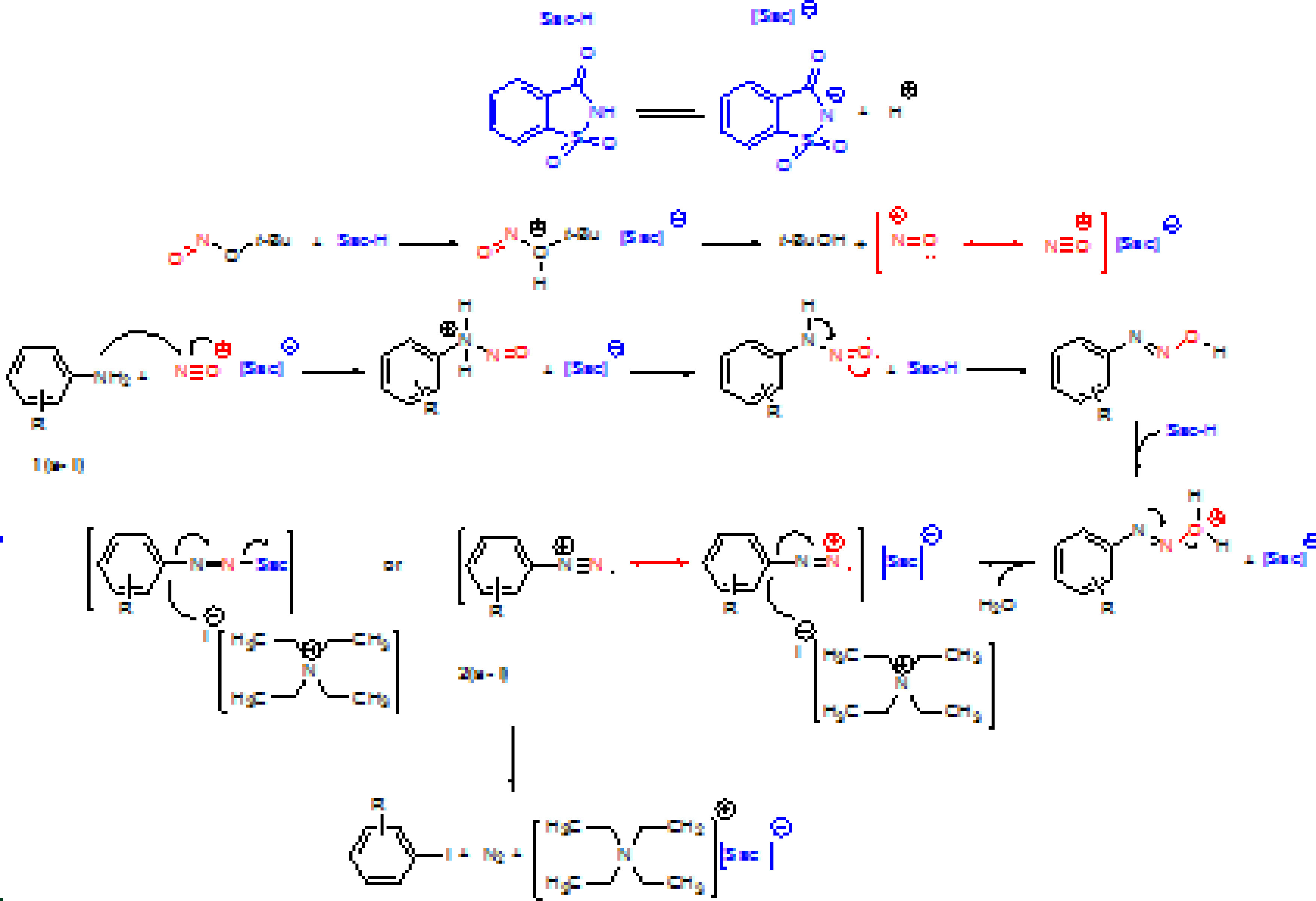

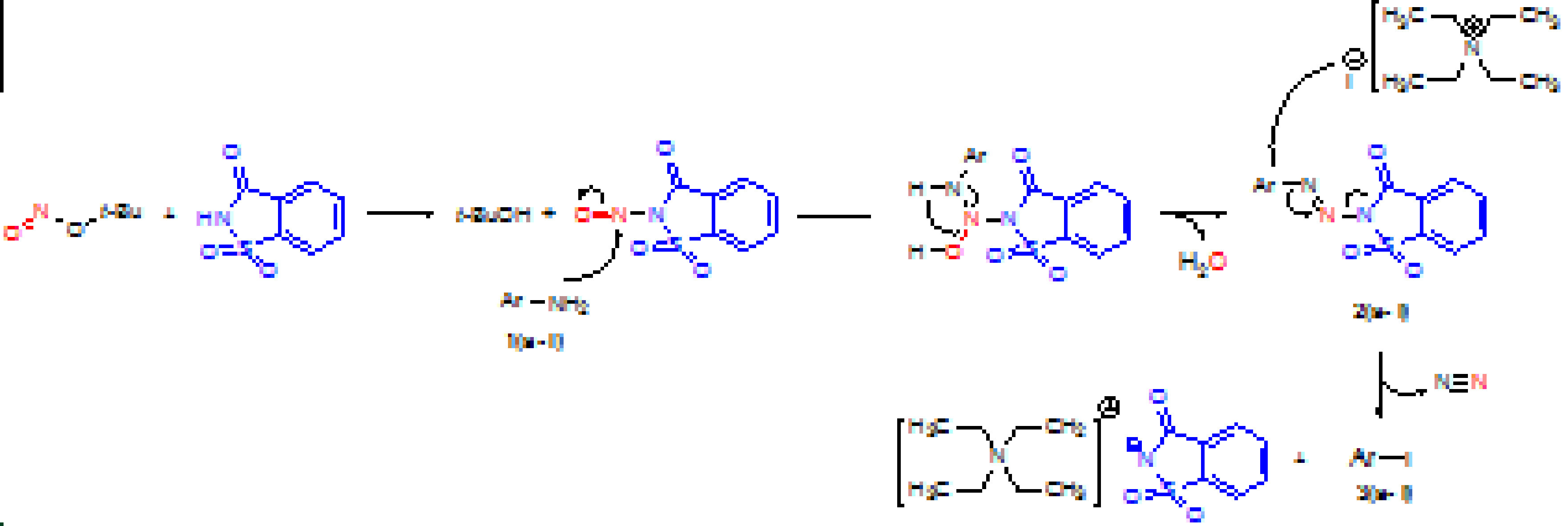
Two proposed mechanisms for the preparation of aryl iodides using TBN and saccharine.

A large scale of the current protocol was also conducted for 1.9 g of 1a under optimal conditions, which afforded 3.12 g of 3a (ca.75%).

Table 2 showed some of the reported methods and their conditions for the preparation of 2-nitro-1-iodobenzene 2k. The previously reported methods have their own merits and limitations, for example, the use of metal-containing nitrites and iodides (Table 2, entries 1, 3–11), low yield and slow rate (Table 2, entries 5,12), 2 steps performance (Table, entries 2,5), use of expensive reagent or strong acids (Table 2, entries 1, 9–11), generation of waste for the preparation of the nitrating reagent (Table 2, entries 5,11), tedious workup (Table 2, entry 12), the formation of toxic and hazard by-products, and incomplete conversions (Table 2, entries 5,7,11).

**Table 2:** Some reported reagents and methods for the diazotization-iodination of 2-nitro aniline 1k.^a^

Entry	mmol of aryl amine	Nitrating agent	Reagents	conditions	Time (min)	Yield (%)	Ref.
1	2	TBN (2.3 mmol)	TFSI–H (2 mmol) HOAc (2.1 mmol) TEAI (2.0 mmol)	EtOH:H_2_O(1:1)/0-5 °C	110	82	4
2	10	IPN (11 mmol)	o−BDSI (12 mmol) glacial AcOH (60 mL) TBAI (10 mmol)	Two steps: Step 1: 0–5 °C; Step 2: CH_3_CN/r.t. °C	45	92	10
3	2	NaNO_2_ (5 mmol)	p-TsOH (6 mmol) KI (5 mmol)	Water-paste form	20-30	72	11
4	2	NaNO_2_ (4 mmol)	sulfonated-resin (5 g) KI (5 mmol)	H_2_O/r.t.	90	71	12
5	5.25	resin-NO_2_(5.25 mmol)	p-TsOH (5.25 mmol) glacial AcOH (8 mL) KI (13.125 mmol)	Two steps: Step 1: H_2_O/r.t.; Step 2: H_2_O/r.t.	30	34	13
6	2	Resin-NO_2_ (6 mmol)	p-TsOH (6 mmol) KI (5 mmol)	H_2_O/r.t.	90	91	14
7	5	KNO_2_ (20 mmol)	HI (20 mmol)	DMSO/35 °C	15	89	15
8	3	NaNO_2_ (6 mmol)	p-TsOH (9 mmol) KI (7.5 mmol)	MeCN/10–20 °C	50	81	16
9	1	NaNO_2_ (2.5 mmol)	[H-NMP]HSO_4_ (4 mmol) NaI (2.5 mmol)	Solvent-free/r.t.	20-30	85	17
10	2	NaNO_2_(4 mmol)	Wet CSA (1.5 g) KI (5 mmol)	Solvent-free/r.t.	12	82	18
11	1	[P4-VP]NO_2_ (0.54 g)	H_2_SO_4_ (2 mmol) KI (2.5 mmol)	Two steps: Step 1: 0–5 °C; Step 2: r.t. or 60 °C	100	74	19
12	10	IPN (30 mmol)	diiodomethane (10 mL)	80 °C	240	65	20
13	2	TBN (2.3 mmol)	Sac–H (2 mmol) HOAc (2.1 mmol) TEAI (2.0 mmol)	EtOH:H_2_O(1:1)/0-r.t. °C	120	78	This work

^a^o-BDSI: o−benzenedisulfonimide, CSA: cellulose sulphuric acid, NMP: N-methyl-2-pyrrolidone, P4-VP: poly(4-vinylpyridine), IPN: i -pentyl nitrite, Sac–H: Saccharin, p- SSA: silica sulphuric acid, TBAI: tetrabutyl ammonium iodide, TsOH: p-toluenesulfonic acid, TFSI–H: bis(trifluoromethylsulfonyl) imide.

## 3. Conclusion

In summary, a telescopic reaction developed for the synthesis of aryl iodides in the presence of TBN, Sac–H, glacial acetic acid, and TEAI. The arene diazonium intermediates could be stored for relatively long periods with little reduce of activity. The current methodology is safe, cost-effective, broad substrate scope, and metalfree. All used reagents are commercially available and inert to moisture and air. Also, the saccharine and tetraethylammonium cation were easily isolated from the reaction mixture which reduces the cost and waste of the current protocol.

## 4. Experimental Section

### 4.1. General

The analytical grade chemicals were provided from Merck and Sigma Aldrich Chemical Companies. The chemicals were used without further purification. Products were characterized by their physical constants such as melting point and 1 H NMR. The purity determination of the substrates and reaction monitoring were accompanied by TLC using silica gel SIL G/UV 254 plates or colour test of azo coupling with 2-naphthol. The 1 H NMR spectra and melting points were recorded with Bruker Avance 400 MHz instrument and Büchi B-545 apparatus in open capillary tubes, respectively.

### 4.2. Typical procedure for the synthesis of aryl iodide

To a stirring mixture of glacial acetic acid (0.12 mL, 2.1 mmol) and TBN (0.30 mL, ~

2.3mmol)inethanol(10mL), Sac − −H(0.37g, 2.0mmol)wasslowlyaddedatlow

temperature (an ice bath). After 5 min, aniline derivative 1(a-l) (2.0 mmol) was added dropwise over 5 minutes into the mixture. The solvent, t -BuOH, and residue TBN were removed under reduced pressure after consuming of aniline derivatives (monitored by a color test of azo coupling with 2-naphthol). Then, 5 mL of 0.4 M TEAI solution was added into stirring as-obtained intermediate 2(a-l) in one portion at low temperature. After confirmation of substrate consumption by a negative test of azo coupling with 2-naphthol, the boiling water was added to the reaction mixture (15 mL) and the aqueous layer was separated. The organic layer was washed with aq. 10% sodium sulphite (3 ×5 mL), then dried over Na2 SO_4_ , filtered, and dried under reduced pressure in a rotary evaporator. The purification was conducted by flash chromatography with n-hexane-EtOAc (9:1, v/v) as eluent.

The recovery of reagents was conducted by adding the concentrated hydrochloric acid into the aqueous layer, and the water evaporated under reduced pressure. Then, the residue was extracted 5times with EtOAc (5 mL), and the collected extracts were dried over anhydrous Na2 SO_4_ and filtrated. The evaporation of solvent afforded Sac–H in 72% yield. The tetraethylammonium chloride (TEAC) was isolated in a yield of 0.25 g from the residue of the above procedure (68%). The melting points and FTIR spectra of recovered saccharin and TEAC were identical to the authentic compound (Sigma Aldrich ≥ 98%).

Physical and 1 H NMR data of 4-methoxybenzenediazonium saccharinate (2b):1 H NMR (400 MHz, DMSO-d6)δ 8.41-8.48 (m, 1H), 8.32-8.39 (m, 2H, ArH), 7.87-7.94 (m, 1H, ArH), 7.29 (d, J = 8.8 Hz, 2H, ArH), 7.03 (d, J = 8.8 Hz, 2H, ArH), 3.80 (s, 3H, OCH_3_) ppm [6].

Spectra data of the aryl iodides 3a-3l [6]

Iodobenzene (3a): 1 H NMR (400 MHz, CDCl3)δ 7.75 (d, J = 8.2 Hz, 2H, ArH), 7.34 (t, J = 7.2 Hz, 1H, ArH), 7.13 (t, J = 7.2 Hz, 2H, ArH) ppm; 13 C NMR (100 MHz, CDCl3)δ 137.7, 130.4, 127.8, 95.0 ppm; EI-MS: M+ m/z 204.

1-Iodo-4-methoxybenzene (3b): 1 H NMR (400 MHz, CDCl3)δ 7.56 (d, J = 8.2 Hz, 2H, ArH), 6.65 (d, J = 8.2 Hz, 2H, ArH), 3.76 (s, 3H, OCH_3_) ppm; 13 C NMR (100 MHz, CDCl3)δ 159.6, 138.4, 116.5, 82.8, 55.5 ppm; EI-MS: M+ m/z 234.

1-Iodo-4-methylbenzene (3c): 1 H NMR (400 MHz, CDCl3)δ 7.59 (d, J = 8.2 Hz, 2H, ArH), 6.94 (d, J = 8.2 Hz, 2H, ArH), 2.32 (s, 3H, CH_3_) ppm; 13 C NMR (100 MHz, CDCl3)δ 136.9, 136.5, 131.4, 91.2, 21.1 ppm; EI-MS: M+ m/z 218.

1,3-Dimethyl-2-iodobenzene (3d): 1 H NMR (400 MHz, CDCl3) : δ 7.20-6.92 (m, 3H, ArH) , 2.42 (s, 6H, 2 ×CH_3_) ppm; 13 C NMR (100 MHz, CDCl3) : δ 141.7, 126.4, 126.2, 123.3, 21.8 ppm; MS (EI) m/z M+ 232.

1,3-Diethyl-2-iodobenzene (3e): 1 H NMR (400 MHz, CDCl3) : δ 7.21 (t, J = 8.0 Hz, 1H, ArH), 7.06 (d, J = 8.0 Hz, 2H, ArH), 2.81 (q, J = 7.0 Hz, 4H, 2 ×CH_2_) , 1.23 (t, J = 7.0 Hz, 6H, 2 ×CH_3_) ppm; 13 C NMR (100 MHz, CDCl3) : δ 147.3, 128.1, 125.9, 107.2, 35.5, 14.7 ppm; MS (EI) m/z M+ 260.

1,3-diisopropyl-2-iodobenzene (3f ): 1 H NMR (400 MHz, CDCl3)δ 7.23 (t, J = 8.0 Hz, 1H, ArH), 7.05 (d, J = 8.0 Hz, 2H, ArH), 3.39 (sept, J = 7.0 Hz, 2H, 2 ×CH), 1.23 (d, J = 7.0 Hz, 12H, 4 ×CH_3_) ppm; 13 C NMR (100 MHz, CDCl3)δ 151.0, 128.3, 123.8, 109.2, 39.4, 23.4 ppm; MS (EI) m/z M+ 288.

1-Chloro-4-iodobenzene (3g): 1 H NMR (400 MHz, CDCl3)δ 7.59 (d, J = 8.2 Hz, 2H, ArH), 7.40 (d, J = 8.2 Hz, 2H, ArH) ppm; 13 C NMR (100 MHz, CDCl3)δ 138.0, 133.8, 130.4, 91.2 ppm; EI-MS: M+ m/z 238.0.

1-Chloro-2-iodobenzene (3h): 1 H NMR (400 MHz, CDCl3)δ 7.82 (d, J = 7.8 Hz, 1H, ArH), 7.44 (d, J = 7.8 Hz, 1H, ArH), 7.28 (t, J = 7.2 Hz, 1H, ArH), 6.97 (t, J = 7.8 Hz, 1H, ArH) ppm; 13 C NMR (100 MHz, CDCl3)δ 141.1, 138.8, 129.6, 128.6, 128.0, 98.6 ppm; EI-MS: M+ m/z 238.0.

1-Bromo-4-iodobenzene (3i): 1 H NMR (400 MHz, CDCl3)δ 7.53 (d, J = 8.2 Hz, 2H, ArH), 7.20 (d, J = 8.2 Hz, 2H, ArH) ppm; 13 C NMR (100 MHz, CDCl3)δ 139.1, 133.4, 122.0, 92.2 ppm; EI-MS: M+ m/z 238.0.

1-Iodo-4-nitrobenzene (3j): 1 H NMR (400 MHz, CDCl3)δ 7.97 (d, J = 8.2, 2H, ArH), 7.89 (d, J = 8.2 Hz, 2H, ArH) ppm; 13 C NMR (100 MHz, CDCl3)δ 147.6, 138.8, 125.2, 102.8 ppm; EI-MS: M+ m/z 249.

1-Iodo-2-nitrobenzene (3k): 1 H NMR (400 MHz, CDCl3)δ 7.91 (d, J = 7.8 Hz, 1H, ArH), 7.84 (d, J = 8.2 Hz, 1H, ArH), 7.49 (t, J = 7.8 Hz, 1H, ArH), 7.30 (t, J = 8.2 Hz, 1H, ArH) ppm; 13 C NMR (100 MHz, CDCl3)δ 153.1, 142.0, 133.5, 129.2, 125.5, 86.3 ppm; EI-MS: M+ m/z 249.

4-(4-iodophenyl)morpholine (3l): 1 H NMR (400 MHz, CDCl3)δ 7.56 (d, J = 9.0 Hz, 2H, ArH), 6.70 (d, J = 9.0 Hz, 2H, ArH), 3.88-3.83 (m, 4H, 2 ×CH_2_(eq)) , 3.13-3.09 (m, 4H, 2 ×CH_2_(ax)) ppm; 13 C NMR (100 MHz, CDCl3)δ 151.0, 138.0, 117.8, 81.9, 66.9, 50.2 ppm; EI-MS: M+ m/z 289.

Caution! The arene diazonium salts are known potentially explosive in the dry state, thus, they must be cautiously stored and handled in the laboratories. Avoid unnecessary heating and mechanical impact, especially when working on a large scale.

Supplementary Materials
1 H NMR spectra copies of the aryl iodides 3a-3l can be found at Supplementary Material.

## References

[1] (1978). The Chemistry of Diazonium and Diazo Groups$,$ Part 1.

[2] (2017). Telescopic synthesis of azo compounds via stable arenediazonium bis(trifluoromethane) sulfonimide salts by using \textit{tert}-butyl nitrite. Dyes and Pigments.

[3] (1987). Iododediazoniation of arenediazonium salts accompanied by aryl radical ring closure. Journal of Organic Chemistry.

[4] (2018). A facile and sustainable protocol to the preparation of aryl iodides using stable arenediazonium bis(trifluoromethylsulfonyl)imide salts \textit{via} the telescopic process. Heteroatom Chemistry.

[5] (2019). Saccharin: a cheap and mild acidic agent for the synthesis of azo dyes via telescoped dediazotization. Green Processing and Synthesis.

[6] (2018). Saccharin and \textit{tert}-butyl nitrite: Cheap and efficient reagents for the synthesis of 1,. Australian Journal of Chemistry.

[7] (2018). Saccharin: an efficient organocatalyst for the one-pot synthesis of 4-amido-cinnolines under metal and halogen-free conditions. Monatshefte für Chemie.

[8] (2020). Recent advances and applications of \textit{tert}-butyl nitrite (TBN) in organic synthesis. Mini-reviews in Organic Chemistry.

[9] (2018). Recently applications of \textit{tert}-butyl nitrite in organic synthesis-. Part I. Current Organic Chemistry.

[10] (1999). Metal-based X-ray contrast media. Chemical Review.

[11] (1979). Ionisation constants of organic acids in aqueous solution. International Union of Pure and Applied Chemistry (IUPAC).

[12] (2012). Artificial sweeteners. Nutrafoods.

[13] (2004). Artificial sweeteners-do they bear a carcinogenic risk?. Annals of Oncology.

[14] (2010). Advances in the chemistry of saccharins: from synthetic novelties towards biologically active compounds. Current Medicinal Chemistry.

[15] (1960). The reaction of saccharin with amines. N-Substituted-3-amino-1.

[16] (2005). Pseudosaccharin amine derivatives: synthesis and elastase inhibitory activity. Pharmazie.

[17] (2005). Efficient one-pot transformation of aminoarenes to haloarenes using halodimethylisulfonium halides generated \textit{in situ}. Canadian Journal of Chemistry.

[18] (2007). A new, one-step, effective protocol for the iodination of aromatic and heterocyclic compounds \textit{via} aprotic diazotization of amines. Synthesis.

[19] (2011). A Simple and effective protocol for one-pot diazotization-iodination of aromatic amines by acidic ionic liquid [H-NMP]HSO$_{4}$ at room temperature. Iranian Journal of Chemistry and Chemical Engineering.

[20] (2012). Green and efficient diazotization-iodination of aryl amines using cellulose sulfuric acid as a biodegradable and recyclable proton source under solvent-free condition. Scientia Iranica.

