# Supplementary material for: Arene diazonium saccharin intermediates: a greener and cost-effective alternative method for the preparation of aryl iodide
Source: Turk J Chem. 2020 Apr 1;44(3):535–42. doi: 10.3906/kim-2002-26 (PMC7706833; doi:10.3906/kim-2002-26)

Supplementary material

Caution! The arene diazonium salts are known potentially explosive in the dry state. Thus, they must be cautiously stored and handled in the laboratories. Avoid unnecessary heating and mechanical impact, especially when working on a large scale.

^{1}H NMR spectra copies of the aryl iodides (400 MHz, $CDCl_3$)

*Iodobenzene (**3a**)*

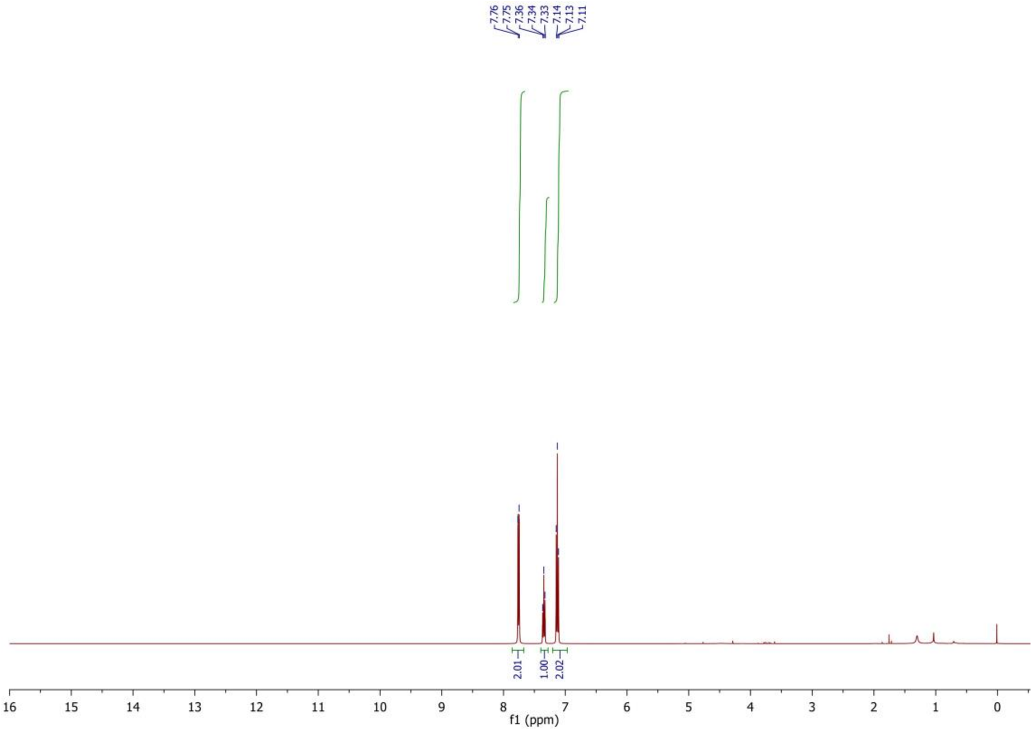

*1-Iodo-4-methoxybenzene (**3b**)*

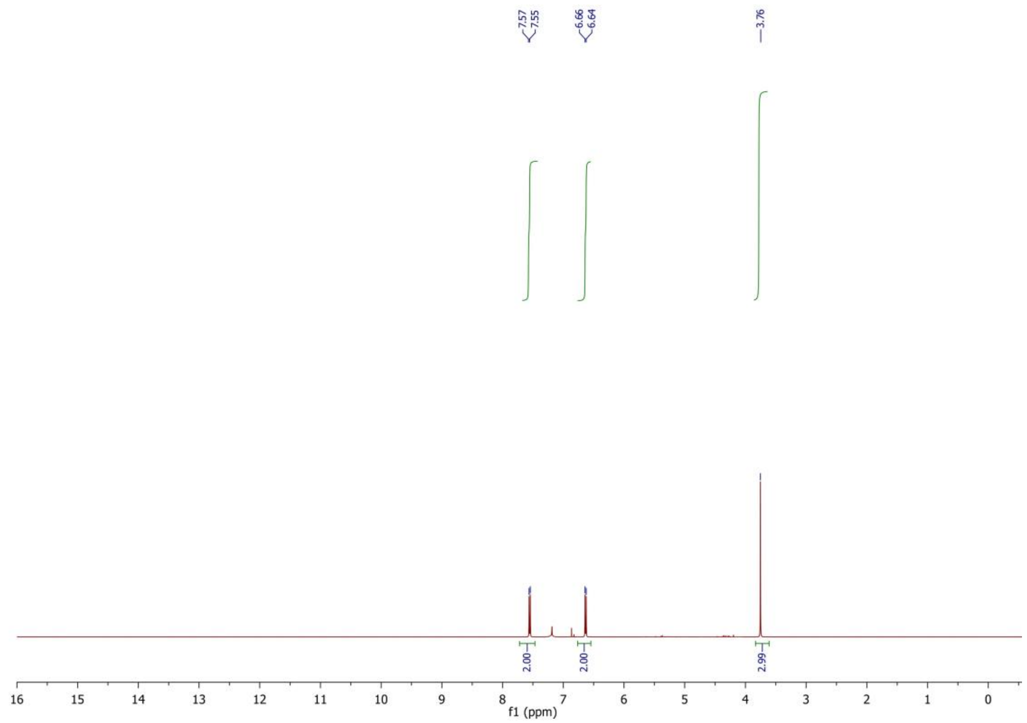

*1-Iodo-4-methylbenzene (**3c**)*

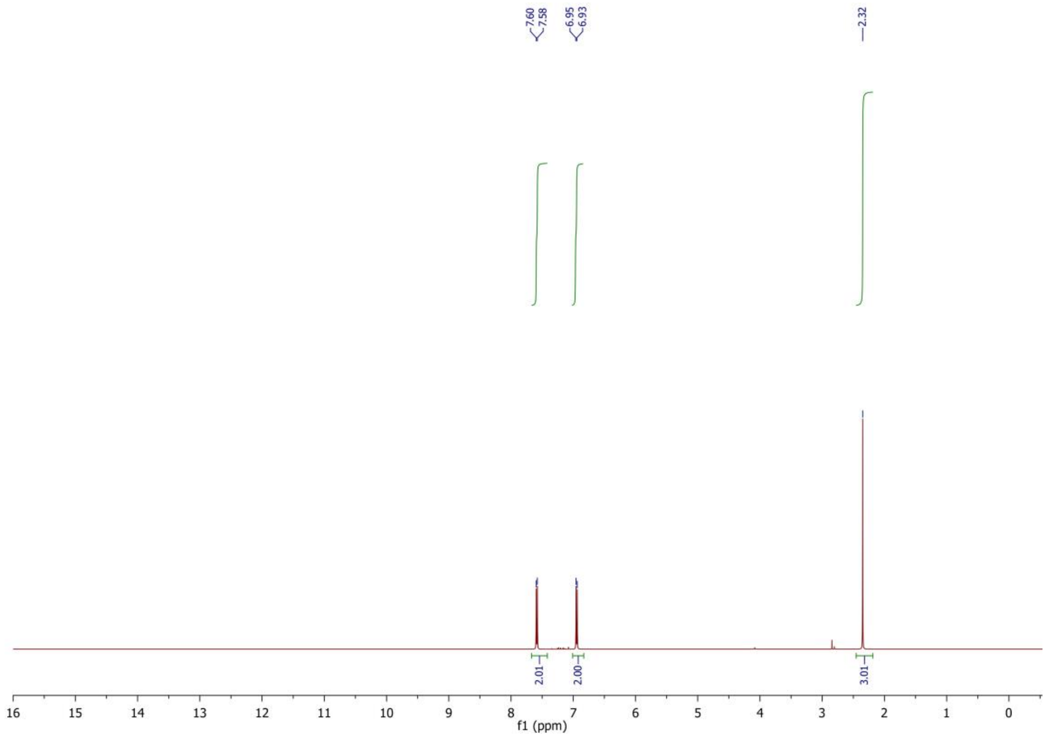

*1,3-Dimethyl-2-iodobenzene (**3d**)*

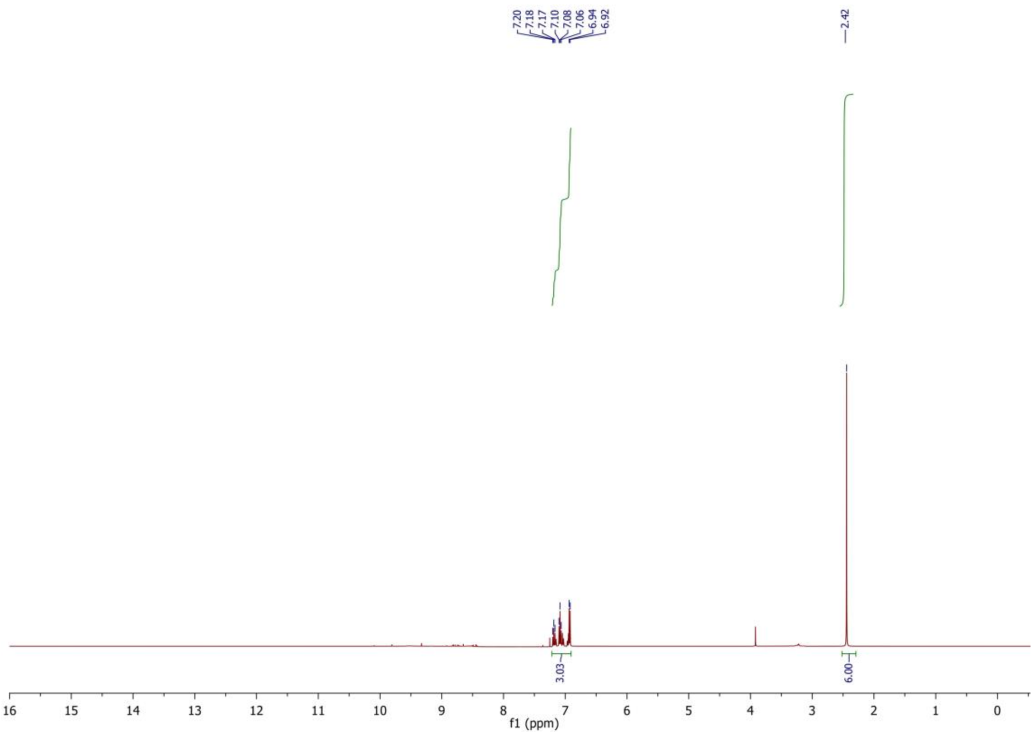

*1,3-Diethyl-2-iodobenzene (**3e**)*

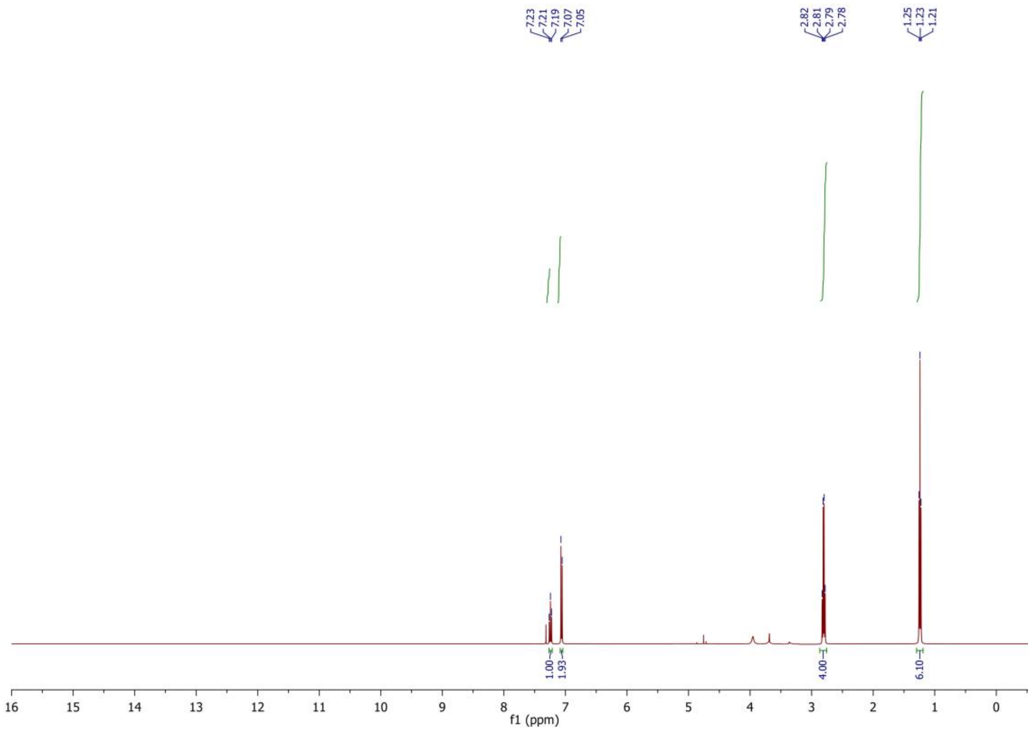

*1,3-diisopropyl-2-iodobenzene (**3f**)*

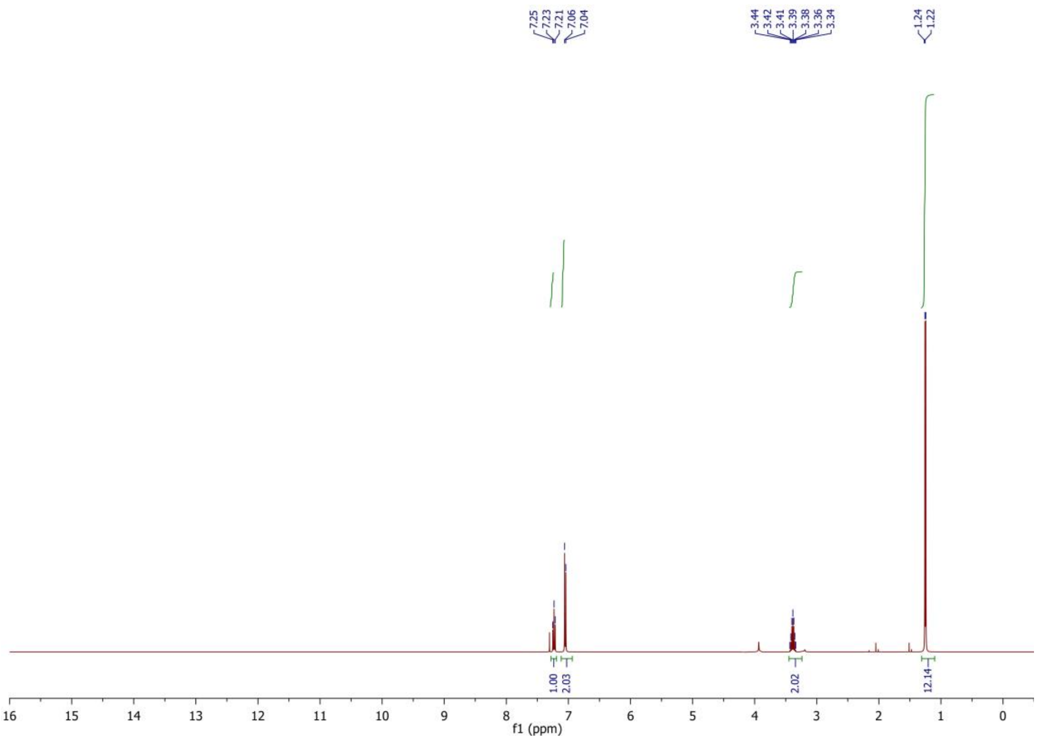

*1-Chloro-4-iodobenzene (**3g**)*

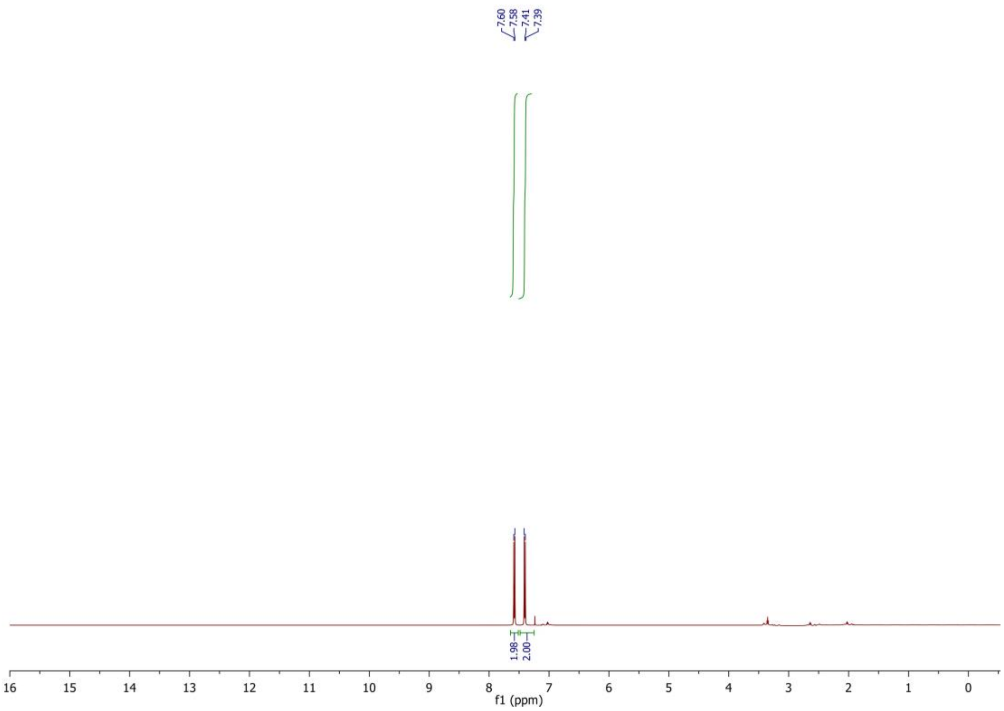

*1-Chloro-2-iodobenzene (**3h**)*

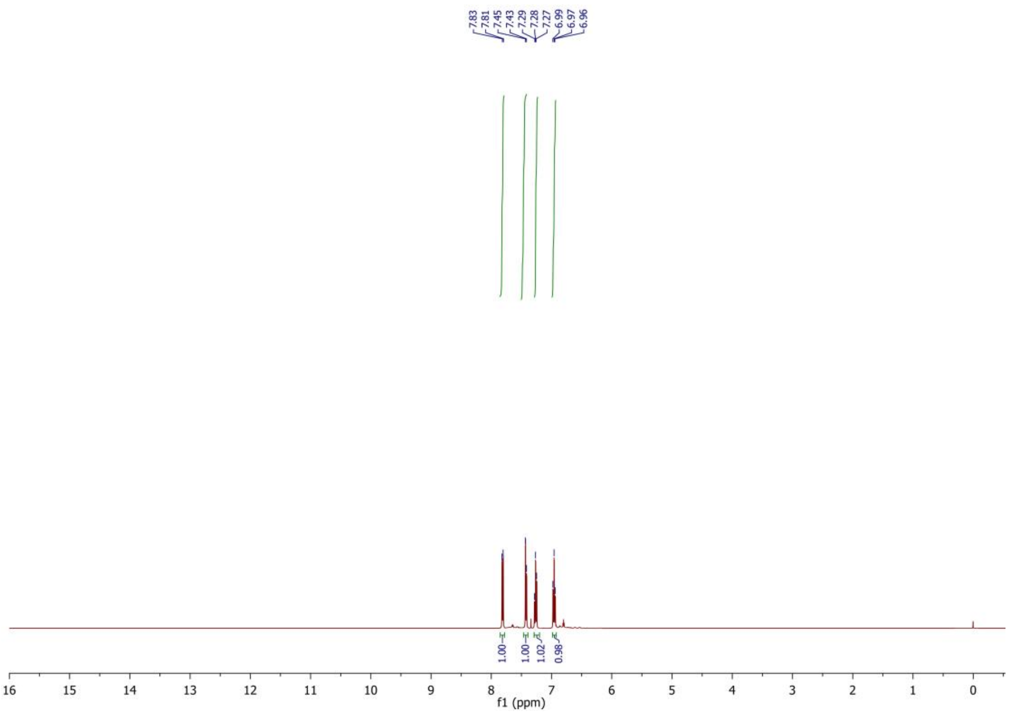

*1-Bromo-4-iodobenzene (**3i**)*

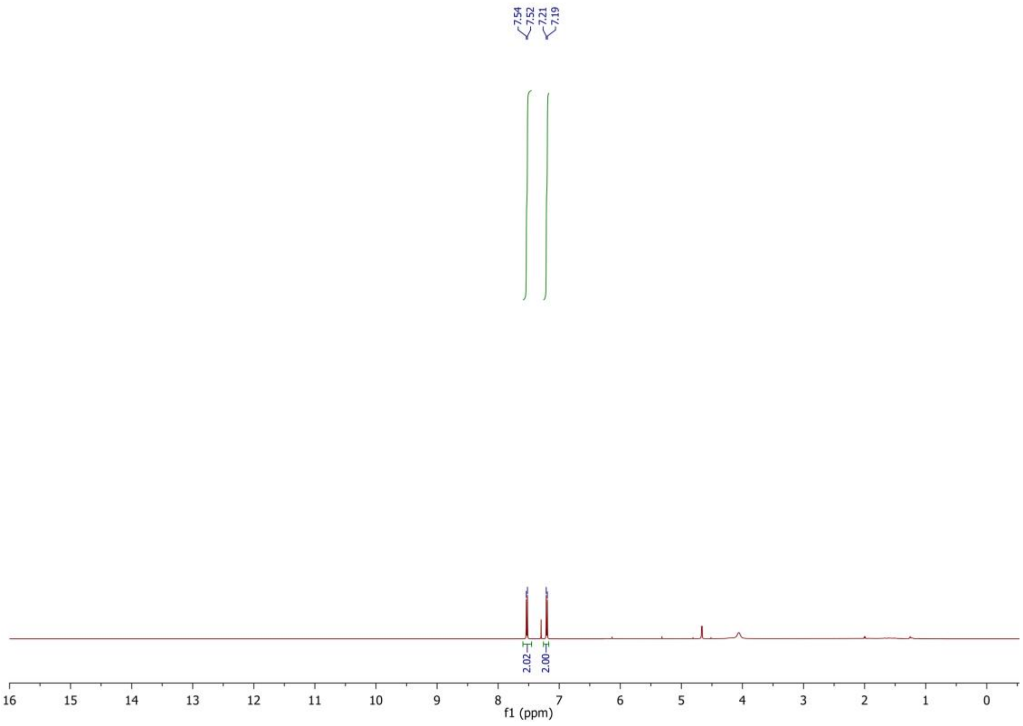

*1-Iodo-4-nitrobenzene (**3j**)*

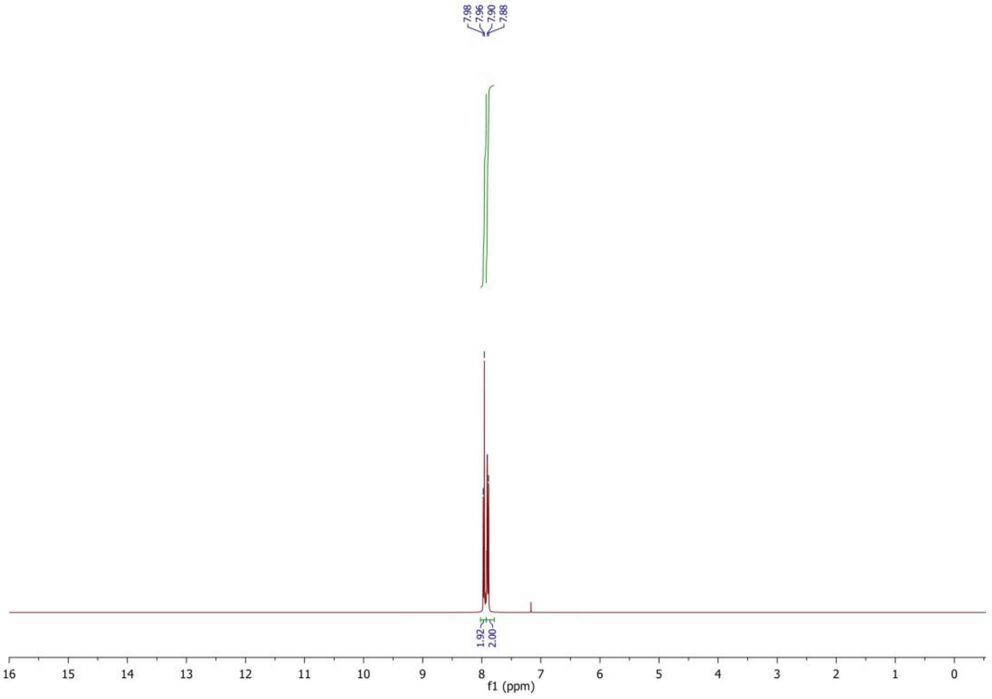

*1-Iodo-2-nitrobenzene (**3k**)*

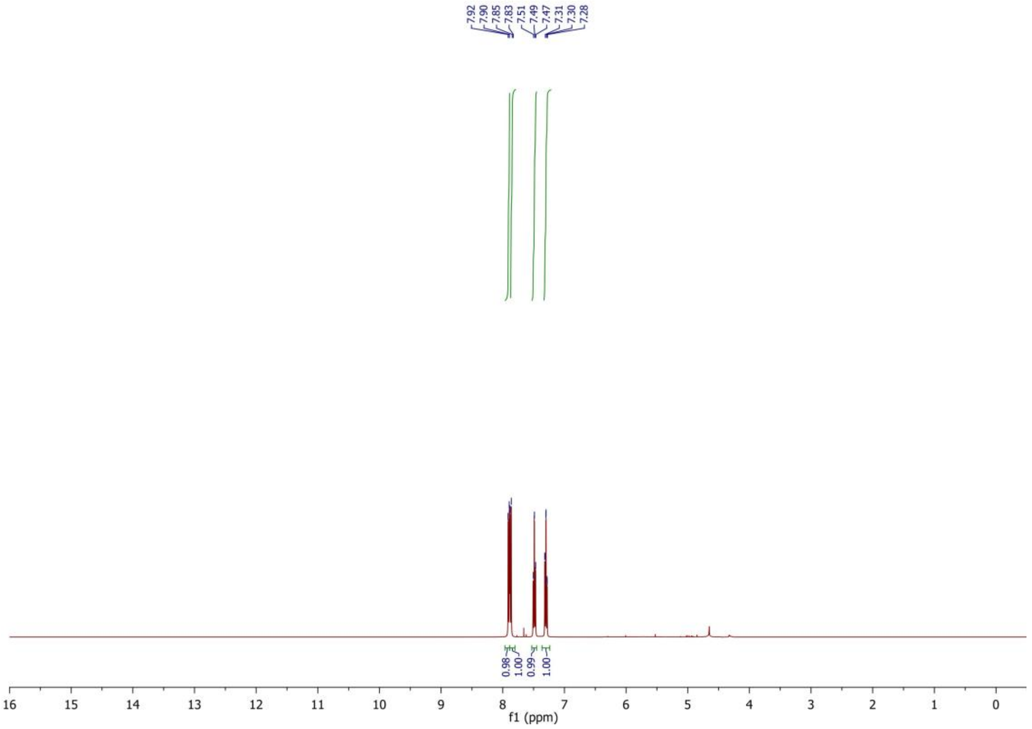

*4-(4-iodophenyl)morpholine (**3l**)*

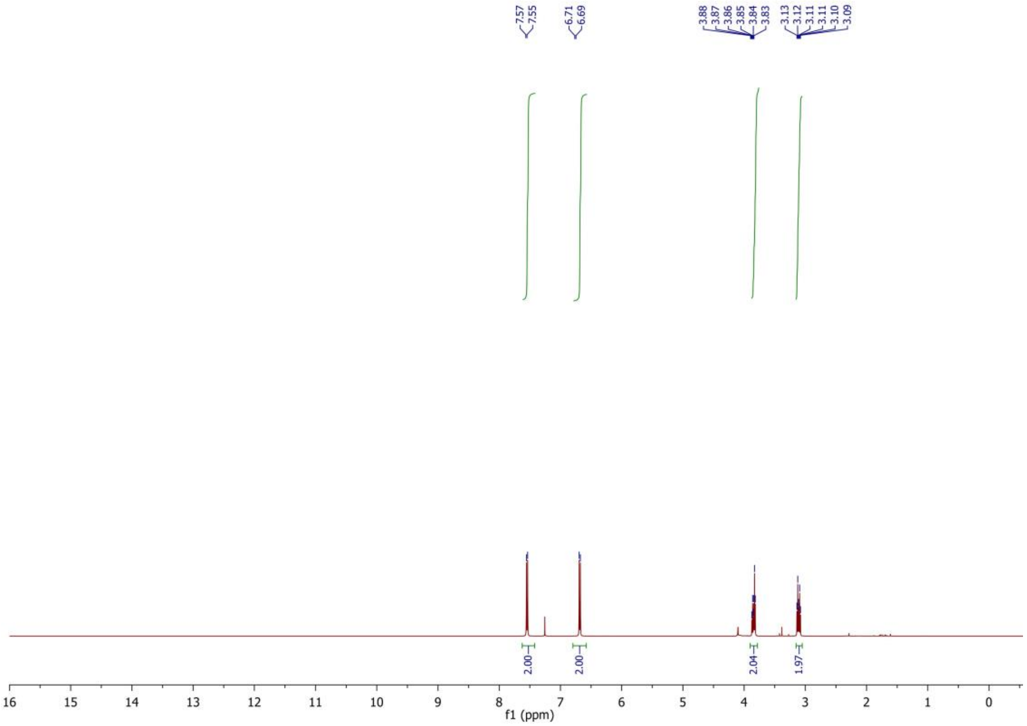

Supplement: Supplementary file 1 — Supplementary Materials 1 H NMR spectra copies of the aryl iodides 3a-3l can be found at Supplementary Material. [file turkjchem-44-535-sup001.pdf]
